# Muscle quality responses to short-term resistance training volume in older adults: an exploratory clinical trial

**DOI:** 10.3389/fragi.2025.1670709

**Published:** 2025-11-20

**Authors:** Kevan S. Knowles, Logan A. Banks, Vanessa C. Cabrera, Wyatt Wilkinson, Adrian J. Pantoja, Isabella G. Thomas, Emily J. Parsowith, Jonathan P. Beausejour, Grant Norte, Jeffrey R. Stout, Matt S. Stock

**Affiliations:** Institute of Exercise Physiology and Rehabilitation Science, University of Central Florida, Orlando, FL, United States

**Keywords:** aging, strength training, echo intensity, ultrasonography, quadriceps, force, longevity

## Abstract

Age-related strength loss is more strongly tied to reduced muscle quality than muscle mass. Echo intensity (EI) measured by B-mode ultrasonography is a common marker of intramuscular adiposity and fibrous tissue. Although high-intensity resistance training is effective, the added value of higher training volumes on muscle quality and strength in older adults remains unclear. Moreover, previous studies examining EI changes following resistance training have provided mixed results. To resolve these issues, this exploratory study compared the effects of different resistance training volumes on muscle quality, size, and strength among older adults. Twenty-five older adults (14 males, 11 females; mean ± SD age = 70 ± 7 years) were randomized to moderate volume (n = 14; 2 sets per exercise; 12 sets per week) or high volume (n = 11; 6 sets per exercise; 36 sets per week) training groups. Cohort-specific test-retest reliability statistics were determined prior to the intervention. Participants trained twice weekly, performing knee extension, trap-bar deadlift, and leg press at 85% of one-repetition maximum (1RM). Pre- and post-intervention assessments included ultrasonographic measures of the vastus lateralis (VL) and rectus femoris (RF), leg lean mass, knee extension 1RM, isometric and isokinetic quadriceps strength, and functional tests. The results indicated no significant group × time interactions with small-to-moderate effect sizes, suggesting that increasing volume three-fold provided no additional benefit. When collapsed across group, improvements were found for VL EI (p = 0.025, η_p_^2^ = 0.200), VL (p = 0.015, η_p_^2^ = 0.232) and RF cross-sectional area (p < 0.001, η_p_^2^ = 0.440), knee extension 1RM (p < 0.001, η_p_^2^ = 0.794), isometric peak torque (p < 0.001, η_p_^2^ = 0.630), and concentric peak torque at 180°/s (p < 0.001, η_p_^2^ = 0.423) and 300°/s (p = 0.009, η_p_^2^ = 0.263). No changes were observed in RF EI, leg lean mass, or functional performance. Despite large mean changes, only <25% of participants exceeded the minimal difference needed to be considered real for any variable. In summary, 6 weeks of moderate volume resistance training elicits meaningful improvements in muscle quality and strength. EI changes were muscle-specific, suggesting heterogeneous adaptations among older adults.

## Introduction

Sarcopenia, the gradual, age-related loss of skeletal muscle mass and strength, will become more prevalent as the global population ages (Cruz-Jentoft et al., 2019). The World Health Organization (WHO) estimates that by the year 2030, the population aged 60 years and older will increase from 1 billion to 1.4 billion, and by the year 2050, this number will double to over 2 billion (WHO, 2022). While aging is strongly related to the development of sarcopenia, lifestyle factors such as low physical activity, sleep deprivation, and poor nutrition may accelerate its development (Tzeng et al., 2020). Sarcopenia can impair functional capabilities, leading to poor outcomes such as falls, fractures, and mortality (Newman et al., 2006; Cruz-Jentoft et al., 2019). Although sarcopenia is often associated with low muscle mass, longitudinal studies suggest that muscle strength declines more rapidly than the loss of muscle mass (Goodpaster et al., 2006; Delmonico et al., 2009). With this divergent trajectory in mind, many have pointed to muscle quality (often defined as strength relative to muscle mass) as a more vital indicator of functional status in older adults (Russ et al., 2012). Delmonico et al. (2009) tracked a large cohort of older adults for 5 years, and over time they demonstrated significant fatty infiltration of midthigh skeletal muscle in both males and females, regardless of subcutaneous thigh adiposity and weight fluctuations. Moreover, strength decreases were found to be up to 5 times greater than the loss of muscle size due to aging (Delmonico et al., 2009). Goodpaster et al. (2008) investigated the impact of a 1-year physical activity program on muscle quality measures in healthy older adults. The non-exercise control group experienced significant decreases in knee extensor strength and increases in thigh intermuscular fat, indicating age-related muscle deterioration. However, the physical activity group did not exhibit these effects, suggesting that the intervention prevented the detrimental changes in muscle quality typically associated with aging. While there is strong evidence to suggest that declines in skeletal muscle quality or composition are associated with poor functional outcomes throughout aging (Rech et al., 2014; Lopez et al., 2017), resistance training is a potent nonpharmacological stimulus that may prevent or even reverse these deleterious effects (Westcott, 2012; Fragala et al., 2019).

Within the last decade, there has been widespread interest in using B-mode ultrasonography-derived echo intensity (EI) to track changes in skeletal muscle quality following resistance training among older adults. The underlying premise of EI as a measurement tool is that skeletal muscle is comprised of both contractile and non-contractile elements, including intramuscular adipose and fibrous tissue, and conducting a gray scale analysis of a specific region of interest can be used to quantify mean pixel intensity, with darker and brighter pixels representing high and low muscle quality, respectively (Pillen et al., 2009; Young et al., 2015). While cross-sectional studies comparing age groups have typically shown worse muscle quality (i.e., higher EI) among older adults (Stock et al., 2020), interventions that have attempted to identify improvements in EI following resistance training have demonstrated mixed results. For instance, Radaelli, et al. (2014a) conducted a detailed, 20-week training intervention among older adults comparing low versus high volume resistance training on measures of skeletal muscle EI, strength, and thickness of the quadriceps muscles. They found that EI of the rectus femoris (RF) significantly decreased for both groups after 13 weeks of training, but only the high volume group showed further improvements at 20 weeks. Following 6 weeks of training, the low volume group experienced a 17.6% increase in knee extension 1RM while the high volume group showed a 22.4.% increase. However, following 20 weeks of training, the low volume group saw a 33.4% increase in 1RM, while the high volume group experienced a 53.3% increase. Moreover, following 20 weeks of training, quadriceps muscle thickness for the high volume group increased by 17.2%, while the low-volume group only increased by 12.6%. These findings suggested that performing additional sets may lead to greater improvements in muscle quality in the long term compared to just 1 set. In another study conducted by Radaelli et al. (2019), they found that after 12 weeks of muscle power training, EI of the quadriceps significantly decreased, regardless of whether 1 or 3 sets per exercise were performed. However, these improvements were small in magnitude when compared to Radaelli, et al. (2014b). This may be due to the differences in loads utilized between the 2 interventions, as Radaelli, et al. (2014a) had participants perform all exercises at a load of 8–20 RM, while Radaelli et al. (2019) utilized 30%–60% of 1RM. Conversely, Scanlon et al. (2014) conducted a 6 weeks lower body resistance training program in older adults to examine the effects of exercise induced changes in EI. While there were significant increases in vastus lateralis (VL) cross-sectional area (CSA), EI remained unchanged in both the VL and RF. Additionally, Yoshiko et al. (2017) conducted a 12 months long resistance training intervention in which they measured EI of the biceps femoris, RF, and VL at baseline, as well as following 6 and 12 months of training. Interestingly, they found that EI improved following 6 months of training but had returned to baseline levels after 12 months of training. The inconsistencies in EI adaptations across studies may be partly attributed to differences in the resistance training protocols employed. Radaelli et al. (2014b) utilized higher training volumes and intensities compared to Scanlon et al. (2014), which could explain the presence and absence of EI improvements, respectively. Furthermore, the use of different muscle groups (quadriceps vs. biceps femoris) and measurement time points (6 weeks vs. 12 months) across studies hinders direct comparisons and may contribute to the mixed findings. Overall, resistance training studies examining changes in EI in older adults have yielded inconsistent results. This discrepancy could be attributed to several factors, including variations in methodological approaches, resistance training protocols, and sample characteristics.

Designing effective resistance training programs for older adults involves careful manipulation of training frequency, exercise selection, intensity, and volume. To induce adaptations in muscle strength and size, guidelines from the National Strength and Conditioning Association recommend performing 1–3 sets of 8–15 repetitions for multi-joint exercises targeting major muscle groups at an intensity of 50%–85% of 1RM, 2–3 times per week (Fragala et al., 2019). In the early phases of resistance training, typically lasting 6–12 weeks, the number of sets performed does not appear to be the primary determinant of muscle strength gains, as several studies have demonstrated that performing either a single set or multiple sets leads to comparable improvements in strength (Abrahin et al., 2014; Radaelli et al., 2014a; Radaelli et al., 2014b). It should be noted, however, that the magnitude of resistance training-induced hypertrophic adaptations are highly variable (Ahtiainen et al., 2016; Stec et al., 2017), a concept known as interindividual response heterogeneity. While individual differences in responsiveness to resistance training exist, higher training volumes across various exercise modalities may be necessary to elicit favorable adaptations among older adults who have difficulty gaining muscle mass, as more robust gains are commonly observed in individuals who are less responsive to training (i.e., “non-responders”) when exposed to higher training volumes (Buford et al., 2013; Montero and Lundby, 2017; Lixandrão et al., 2024). Recently, Lixandrão et al. (2024) used an elegant study design to demonstrate this concept. Using a within-subject unilateral design where 1 leg performed 1 set and the contralateral leg performed 4 sets of knee extension exercise for 10 weeks, the authors demonstrated that increasing resistance training volume mitigated non-responsiveness by improving whole-muscle tissue hypertrophy and strength gains among non-responders. For responders, higher resistance training volume tended to further enhance hypertrophic adaptations, but 1RM strength increased similarly irrespective of training volume. While these findings suggest that resistance training volume manipulation may be an effective strategy for optimizing muscle adaptations in older adults, the current literature has several limitations. First, most studies have focused on measures of muscle size and strength, with less attention given to muscle quality indices like EI. Second, there is a lack of research directly comparing the effect of different training volumes on quadriceps EI in older populations. Consequently, it remains unclear whether higher training volumes are necessary for improving muscle composition and function in this age group.

Despite the evidence supporting the benefits of higher training volumes for muscle hypertrophy, particularly among non-responders (Lixandrão et al., 2024), the optimal resistance training strategies for enhancing muscle quality in older adults remain unclear due to inconsistent findings in EI adaptations. Plausibly, distinct, individualized training strategies may be necessary to improve muscle strength, mass, and quality among older adults, as each of these attributes has unique mechanistic underpinnings. To address these knowledge gaps, the present exploratory study aimed to compare the effects of 6 weeks of moderate versus high volume resistance training on multiple measures of muscle strength, mass, and quality in older adults.

## Methods

### Experimental design

**FIGURE 1:**
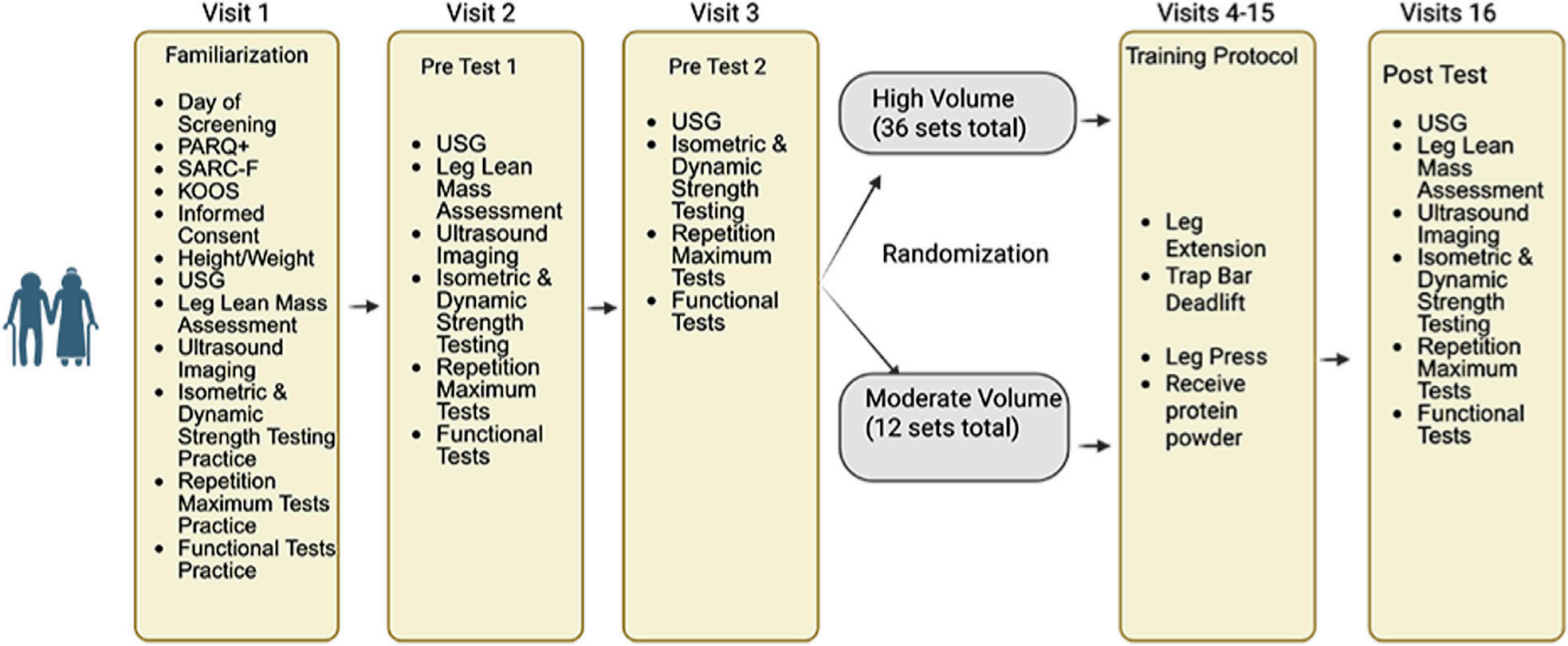
An overview of this study’s design. Created with Biorender.com.

This exploratory study utilized a 6-week, pretest/posttest design in which older adults were randomly assigned to moderate versus high training volume to evaluate changes in multiple measures of skeletal muscle quality (Figure 1). Participants were randomized to one of two groups, based on a randomization Excel spreadsheet that was blocked for sex and group using Claude AI (Anthropic, 2024). Following thorough screening and enrollment, participants completed a battery of tests prior to the start of the resistance training protocol. On each day of testing, participants arrived at the laboratory following a ≥4 h fast and immediately had their hydration status assessed via a urine sample using a digital refractometer (MASTER SUR/NM, ATAGAO USA, Bellevue, WA, United States). Urine specific gravity values ≤1.020 were required to commence testing. Participants completed a battery of tests and assessments in the following order: assessment of leg lean mass, ultrasound measurements of their dominant leg (based on kicking preferences), 5 time-sit-to-stand, and timed-up-and-go (TUG), unilateral isometric and concentric isokinetic strength testing, and 1RM (knee extension) and 5RM testing (trap bar deadlift and leg press). Once enrolled, participants were randomly assigned to one of two groups: moderate volume or high volume resistance training. Both groups completed supervised training sessions twice per week at the University of Central Florida (UCF), scheduled at the same time of day (±1 h), with at least 48 h between sessions. If a training session was missed, participants were permitted to reschedule, provided that no more than 1 week elapsed between consecutive sessions. To reduce learning effects, all participants first completed a familiarization visit, during which they were introduced to the testing procedures and resistance exercises. Investigators provided detailed demonstrations for each test and exercise prior to practice. After the familiarization session, participants returned for two separate pretesting visits to establish baseline measurements. The results from these visits were used to calculate sample-specific test-retest reliability data (i.e., intraclass correlations [ICCs], standard error of measurement [SEM], minimal difference needed to be considered real [MD]), which were used to quantify the effectiveness of the interventions on an individual participant basis. Testing sessions were conducted at the same time of day (±1 h) to minimize diurnal effects. A minimum of 48 h was required between the final resistance training session and posttesting. Among participants who completed the study, only three (two in the moderate volume group and one in the high volume group) completed their posttest exactly 48 h after the final training session. For all other participants, posttesting occurred between 4 and 7 days following the final training session. All resistance training sessions were supervised by study investigators experienced in resistance training techniques.

### Participants

**FIGURE 2:**
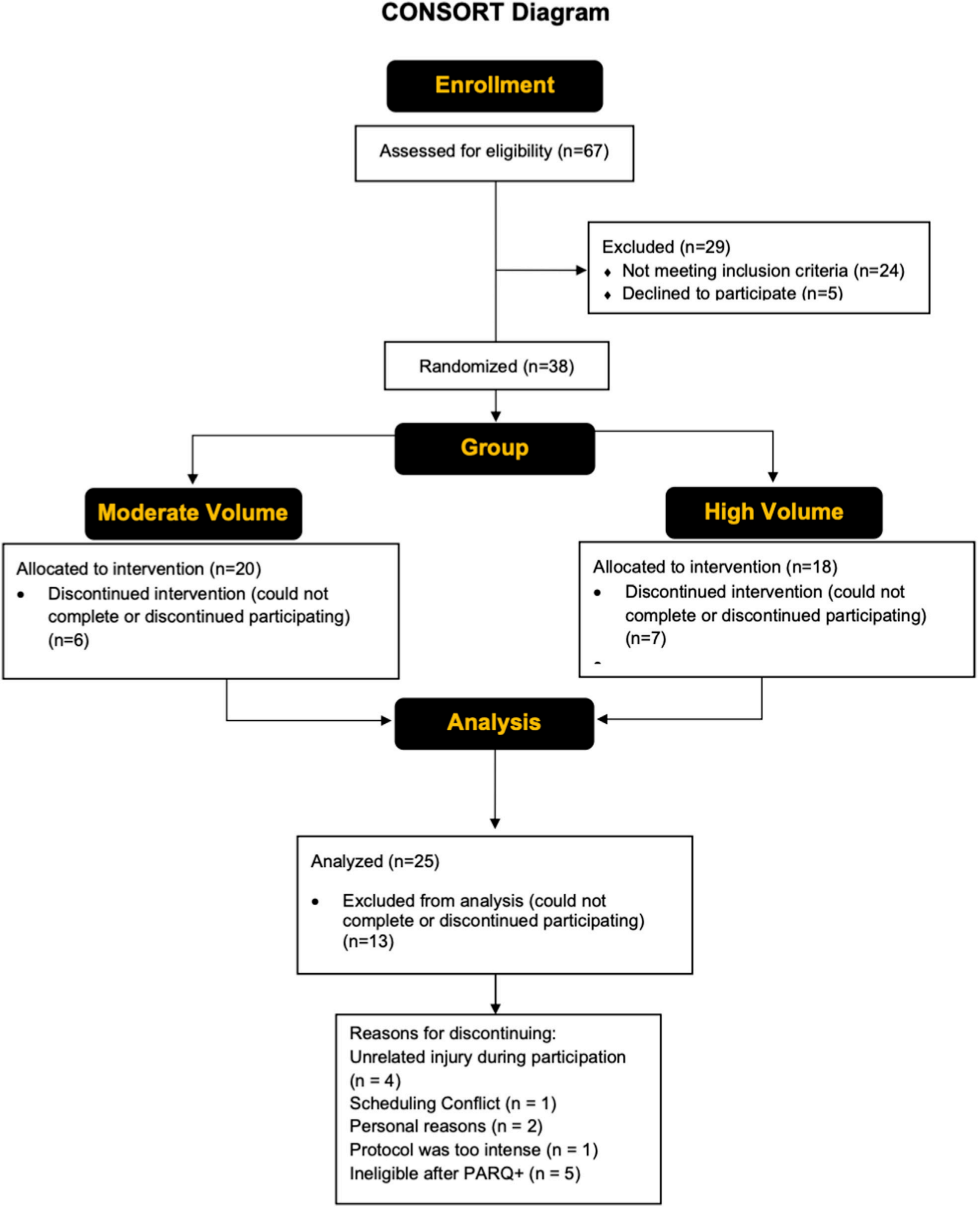
A CONSORT flow diagram showing the enrollment, group allocation, and analysis of the study participants.

Figure 2 shows a CONSORT diagram, providing details of all study participants. Twenty-five older adults (14 males, 11 females; mean ± SD age = 70 ± 7 years, body mass index [BMI] = 24.5 ± 3.1 kg/m^2^) completed the study. Out of the 25 participants, 14 (6 males, 8 females, age = 70 ± 7 years, BMI = 25.6 ± 2.8 kg/m^2^) were randomly assigned to the moderate training group, whereas 11 (7 males, 4 females, age = 70 ± 7 years, BMI = 3.7 kg/m^2^) were randomly assigned to the high volume training group. Recruitment was conducted through the community and the Learning Institute for Elders at UCF group with the use of flyers, word of mouth, social media, and posting of a study link on the UCF College of Health Professions and Sciences research page. Initial screening was conducted either through a phone call or in-person to determine eligibility based on the inclusion criteria of the study. Individuals who had lower-body surgery or used an assistive walking device within the previous year did not qualify. Further exclusion criteria included a history of cancer, neuromuscular and/or metabolic diseases, along with individuals who have experienced a myocardial infarction within the past year. Participants did not qualify if their primary care physician advised them to not participate in exercise and/or if they answered “YES” to any questions on the Physical Activity Readiness Questionnaire (PAR-Q+). The use of prescription medications and dietary supplements was reviewed on a case-by-case basis; however, those that cause muscle weakness were excluded. Outside of the study, participants were asked to maintain their usual physical activity levels (i.e., walking, running, sports, etc.) and dietary habits, but to not begin a new exercise or physical activity regimen. Participants enrolled must have refrained from lower-body resistance training 6 months prior (on average, ≥ once/week). Participants with lactose intolerance were excluded since consumption of whey protein was required (details described below). The study was approved by the UCF Institutional Review Board (#6929), and all participants signed informed consent documents prior to study participation.

### Assessment of sarcopenia and knee injury and osteoarthritis score (KOOS)

Each participant completed the Strength, Ambulation, Rising from a chair, Stair climbing and history of Falling (SARC-F) questionnaire prior to testing. The SARC-F is a five-item questionnaire that can predict/screen individuals who are at risk of sarcopenia (Malmstrom et al., 2016). The SARC-F consists of 5 components: strength, assistance walking, rise from chair, climb stairs, and falls. Scoring for the SARC-F is based on a 0–10 scale (0 = best; 10 = worst) with each component being worth between 0 and 2 points (0 = no difficulty; 1 = little to some difficulty; 2 = lots of difficulty/require aid). A score equal to or greater than four is predictive of sarcopenia and poor outcomes. Following the completion of the SARC-F, participants completed the KOOS survey which is a 42 item questionnaire that evaluates short and long term symptoms and function with those with knee injuries and osteoarthritis (Roos and Lohmander, 2003). The SARC-F and KOOS data were used in this study for descriptive purposes, rather than as key outcomes.

### Assessment of leg lean mass

Bilateral leg lean mass and dominant leg lean mass (kg) were measured at both pre and post testing using an Inbody 770 multi-frequency bioelectrical impedance (BIA) device (Inbody 770, Biospace Co. Ltd. Seoul, Korea). Participants stood on the platform of the device barefoot with the soles of their feet on the electrodes and grasped the handles of the device to maintain contact with electrodes. Participants remained still for 1 min while keeping their elbows fully extended and their shoulder joints abducted to a ∼30° angle. Leg lean mass measurements were obtained from the Inbody 770 multi-frequency BIA using its built-in algorithms and software.

### Assessment of muscle size and quality

A portable B-mode imaging device and a multi-frequency linear-array probe (GE Logiq e BT12, GE Healthcare, Milwaukee, WI, United States) were used to obtain ultrasonography images of the dominant RF and VL. To capture images of the VL, participants were instructed to lie on the non-dominant side of the body with their legs on top of each other, with their knee joint positioned at an angle of 10°, on a treatment table. Participants rested in the supine position for acquisition of images from the dominant RF. Ultrasonography images of both muscles were captured in the transverse plane using the panoramic function. Images of the VL were taken at a location corresponding to 50% of the distance between the greater trochanter and superior patella. For the RF, images were taken at a location corresponding to 50% of the distance between the anterior superior iliac spine and superior patella. A high-density foam pad was secured around the limb to ensure stable probe movement in the transverse plane. Ultrasound settings (frequency: 12MHz, gain: 50 dB, dynamic range: 72) were kept consistent across all participants. Image depth was set to 5.0 cm, but increased on a case-by-case basis in the event that the entire muscle belly was not visible. A generous amount of water-soluble transmission gel (Aquasonic 100 ultrasound gel, Parker Laboratories, Inc., Fairfield, NJ, United States) was applied to the skin so that the probe surface was immersed during testing to enhance acoustic coupling. Three images were taken for each participant; however, a single image where the muscle borders were most well defined was utilized for analysis. The same investigator captured ultrasonography images (K.S.K).

**FIGURE 3:**
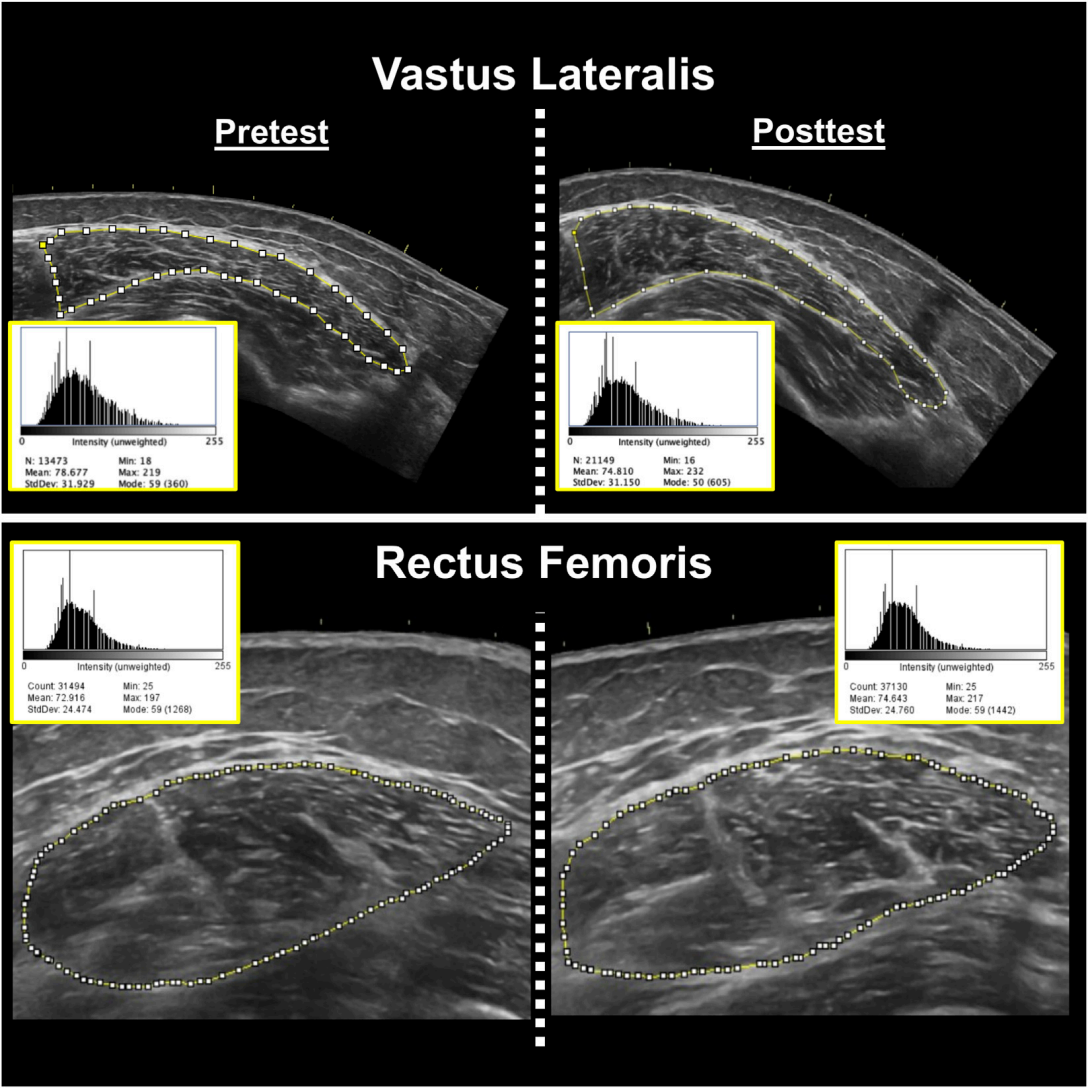
Representative pretest (left) and posttest (right) B-mode ultrasound images of the vastus lateralis (top) and rectus femoris (bottom) muscles from two different participants. Corresponding ImageJ grayscale histograms are shown adjacent to each image, with mean pixel intensity values representing muscle echo intensity. Higher mean values indicate greater intramuscular echogenicity (i.e., reduced muscle quality).These values do not account for subcutaneous adipose tissue thickness. The same investigator conducted all analyses, showed excellent intrarater reliability prior to data collection, and was blinded during analysis.

Following data collection, the images were analyzed using ImageJ (ImageJ, version 1.51, NIH, Bethesda, MD, United States). The same investigator responsible for image acquisition (K.S.K) conducted image analysis but was blinded to participant and testing date during image analysis. The variables of interest were muscle CSA (cm^2^) and corrected EI. The polygon function was used to quantify muscle CSA, with the borders of each muscle carefully traced in ImageJ. All EI was assessed by computer-aided gray-scale analysis using the histogram function. The EI values were determined as the mean value of the pixel intensity histogram, with values ranging between 0 and 255 arbitrary units (A.U.) (black = 0; white = 255). Subcutaneous adipose tissue thickness (cm) over each muscle was measured using the straight-line function, and corresponded to the mean of left, center, and right values. The EI values were corrected for subcutaneous adipose tissue thickness using an equation created by Young et al. (2015). Prior to the study, the investigator involved in image acquisition and analysis demonstrated excellent intrarater reliability for CSA (VL ICC_3,1_ = 0.977, SEM = 7.14%; RF ICC_3,1_ = 0.988, SEM = 4.34%) and EI (VL ICC_3,1_ = 0.993, SEM = 1.86%; RF ICC_3,1_ = 0.999, SEM = 0.56%) among 10 healthy adults. Example ultrasound images have been shown in Figure 3.

### Sit to stand functional assessment

The sit-to-stand chair stand test was used to assess lower body strength (Bohannon et al., 2010). At the start of the test, participants sat on a chair with their arms crossed. They were instructed not to sit back in the chair, ensuring that their back did not rest on the back pad of the chair. Participants were then instructed to stand up from the chair and sit back down for a total of 5 repetitions, while keeping their arms crossed across their chest and legs shoulder-width apart. Participants were instructed not to touch the back of the chair throughout the set. Time to complete 5 repetitions from the first stance to final sit was recorded in seconds with a digital stopwatch (A601X-Pro Survivor Stopwatch, ACCUSPLIT, Pleasanton, CA, United States).

### Timed up and go functional assessment

The TUG test was performed to assess balance and functionality (Podsiadlo and Richardson, 1991). The TUG began with participants standing up from a chair and walking along a marked line on the floor for a short distance (3 m), turning around to return to the chair and sitting back down on the chair. The timing began the moment the participant stood up from the chair and ended once they were seated again. After an initial practice trial, participants completed 3 timed trials at a comfortable walking pace. Time trials were recorded in seconds with the use of a Dashr React Timing System (Dashr Inc., Lincoln, NE, United States).

### Assessment of isometric peak torque and concentric peak torque

Maximal voluntary contractions of the dominant knee extensors (based on kicking preference) were performed with participants seated on a Biodex System 4 isokinetic dynamometer (Biodex Medical Systems, Shirley, NY, United States). Participants were seated and restrained with straps around their chest, hips, thigh, and ankle. Isometric strength testing was conducted with hip and knee angles set at 95° and 90°, respectively. Before testing, participants performed a brief submaximal warm-up consisting of 3-s contractions at progressively greater intensities up from 50%, 70%, and 90% of their perceived maximal effort. After the warm-up, participants performed 3 maximal voluntary contractions (MVCs), separated by 2 min of rest. Following the assessment of isometric knee extensor peak torque, participants performed 3 maximal concentric isokinetic muscle actions of the knee extensors at velocities of 180° and 300°/second, with the testing velocities separated by 2 min. Participants received strong verbal encouragement and were instructed to kick out “as hard and fast as possible” during all testing on the Biodex isokinetic dynamometer. For all isometric and concentric muscle actions, peak torque was quantified with software installed on the Biodex isokinetic dynamometer desktop computer (Biodex Advantage, Biodex Medical Systems, Shirley, NY, United States). Peak torque has been reported both in absolute units (Nm) and relative to dominant leg lean mass (Nm/kg).

### Assessment of 1RM and 5RM strength

Prior to beginning the resistance training program, dynamic maximal strength was determined by performing a 1RM for the knee extension. The 1RM protocol was adapted using guidelines from Sheppard and Triplett (2015). On the day of testing, participants completed 2 warm-up sets with a very light load for 5–10 repetitions, followed by 3–5 min rest between each set. The load was then increased an additional 10%–20% and participants were instructed to attempt to complete 5 repetitions. Participants who were able to successfully complete the first 1RM attempt with proper technique were given 3–5 min rest before performing another 1RM attempt with the load increased an additional 4–8 kg. The 1RM test was completed once the participant was able to achieve a true 1RM, with proper form, within 5 attempts. If a participant had failed a 1RM attempt, the load from the previous attempt was recorded as the 1RM. Changes in 1RM were analyzed both in absolute terms (kg) and relative to bilateral leg lean mass (Nm/kg).

Prior to the start of the training intervention, 5RM strength tests were conducted for both the trap bar deadlift and leg press exercises. The testing procedures closely mirrored those used to determine the 1RM for the knee extension, as previously described. The primary purpose of the 5RM assessments was to establish appropriate initial training loads for each participant in a manner that ensured both safety and effective exercise prescription. These 5RM values were not intended to serve as outcome measures and were therefore not included among the study’s dependent variables.

### Resistance training

Upon enrollment, participants were randomly assigned to either the moderate or high volume group. Each participant performed supervised resistance training twice per week for 6 weeks (i.e., 12 total resistance training sessions). Participants assigned to the moderate volume group performed 2 sets of the knee extension, trap bar deadlift, and leg press (i.e., 6 total sets/session), whereas those in the high volume group performed 6 sets of these exercises (i.e., 18 total sets/session). The training goal was for each set to be performed to volitional failure with the equivalent of a 5RM load, which was initially assessed during the second pretesting visit according to guidelines described by the National Strength and Conditioning Association (Sheppard and Triplett, 2015). Progressive overload was ensured by adding weight to each exercise throughout the duration of the study. Training loads were adjusted on a set-by-set basis. The goal for each set was to perform between 3 and 7 repetitions. For example, if a participant was able to complete 2 repetitions for any given set, weight was removed prior to the next set. On the contrary, if a participant performed 8 repetitions for any given set, more weight was added for the next set. This approach has been proven successful for inducing rapid gains in strength in short term studies (DeFreitas et al., 2011; Stock et al., 2017). A rest period of 2–3 min was allowed between all sets and repetitions. Participants performed each exercise with a full range of motion and were cued to “go as fast as possible” on the concentric action of each exercise. A single warmup set of 5–10 repetitions was performed prior to each exercise with a load corresponding to ≤50% of the participants’ estimated 1RM.

### Nutrition analysis

Each participant was given a hand-written food log during the last week of training that required them to track all food and beverages consumed. They were asked to record a total of 3 days and were instructed to pick 2 weeks days and 1 weekend day to record their food and beverage intake. Participants were instructed to log every item as specifically as possible with item brand names, method of preparation, and measurement of food/beverage quantities if feasible. Participants were informed to keep their diet and caloric intake consistent throughout the study. Investigators also requested that participants be consistent with caffeine consumption. Investigators reminded the participants often about logging in to their food logs during their training sessions. Food logs were collected on training visits. Total caloric intake, protein, carbohydrates, and fats were analyzed from each food log using an online software (MyFitnessPal, Inc., San Francisco, CA, United States).

To support the participants’ protein intake, two bags of whey protein powder (Outwork Nutrition, Inc., Orlando, FL, United States) were given to them at the start of the training visits. They were instructed to consume 2 scoops of protein (∼50 g of protein) on training days, and 1 scoop (∼25 g) on non-training days (Deutz et al., 2014). The protein content from the provided whey supplement was included in the reported caloric and macronutrient totals.

### Physical activity

To assess whether habitual activity differed after the assigned interventions, daily physical activity for each participant was objectively measured during the last week of the training via ActiGraph GT×9 Link (ActiGraph, Pensacola, FL, United States) accelerometers. The accelerometers were programmed to record acceleration in 60 s periods with low frequency extension (Kim et al., 2015). Participants were instructed to wear the accelerometers on the right side of the waist, attached via an elastic belt. The accelerometers were worn during all waking hours (except water-based activities) for 7 consecutive days for a duration of 1 week. A valid day was considered as having ≥10 h of monitor wear time (Troiano, 2007). All data were processed using ActiLife version 6.13.3 software. All data points were entered into a wear-time validation program to determine if participants met the required amount of wear time during the monitoring period. The validation program also identified any periods of non-wear time, defined as ≥ 60 min of zero counts, which were then excluded from the analysis (Troiano, 2007). To determine physical activity levels, assessment of counts per minute (CPM) was utilized (Troiano, 2007). Activity counts were averaged into 60-s epochs and categorized using a validated algorithm (Troiano, 2007) to determine time spent in moderate (2020–5998 CPM) and vigorous (≥5999 CPM) physical activity. Minutes of moderate-to-vigorous physical activity (MVPA) and total daily step counts were averaged across each valid wear day.

### Statistical analysis

This study employed multiple statistical approaches. First, data from the two pretest visits were used to assess test-retest reliability following the guidelines outlined by Weir (2005). For each dependent variable, the ICC (model 3,1) and SEM were calculated, with SEM values reported in both absolute units and as a percentage of the grand mean. Paired samples *t*-tests were also conducted to evaluate systematic differences between the two pretest sessions. MD values were calculated for each group to determine the smallest change considered meaningful, also based on the recommendations of Weir (2005). Before analyzing changes in response to the interventions, Shapiro-Wilk tests were conducted to assess normality, and independent samples *t*-tests were used to evaluate baseline differences between groups. When assumptions for normality and baseline equivalence were met, each dependent variable was analyzed using a two-way mixed factorial analysis of variance (ANOVA) with time (pre, post) as the within-subjects factor and group (moderate volume, high volume) as the between-subjects factor. When a significant time × group interaction or main effect was observed, Bonferroni-corrected *post hoc* pairwise comparisons were conducted. In instances where key assumptions for ANOVA were violated−such as unequal baseline values−an analysis of covariance (ANCOVA) was employed, using pretest scores as a covariate and posttest values as the dependent variable. Partial eta squared statistics (η_p_^2^) were used as a measure of the effect size for each ANOVA, with values of 0.01, 0.06, and 0.14 representing small, medium, and large effects, respectively (Cohen, 1988). An alpha level of 0.05 was used to determine statistical significance. In addition, we evaluated change scores on an individual participant basis to determine the number of participants that showed changes exceeding the MD. After confirming normal distributions and homogeneity of variance, independent samples *t*-tests were used to compare caloric and macronutrient intakes and physical activity data between groups. JASP (version 0.18.3, University of Amsterdam, Amsterdam, Netherlands) was used to conduct all statistical analyses with the exception of the reliability analyses, which were conducted with a custom Excel spreadsheet (Microsoft Corporation, Redmond, WA, United States).

## Results

### Test-retest reliability

Test-retest reliability statistics can be found in Table 1. The analysis demonstrated excellent reliability (ICC >0.88) for most strength, body composition, and ultrasound measures, including 1RM knee extension, leg lean mass, and corrected echo intensity. Functional performance measures like TUG and sit-to-stand also showed good reliability, though with slightly greater variability. Notably, 1RM knee extension showed a significant increase at retest, likely due to neuromuscular learning and/or rapid strength adaptations.

**TABLE 1:** Test-retest statistics for each of the dependent variables in this study. The SEM and MD are in the same units of measurement as the given dependent variable.

Dependent Variable	Test (mean ± SD)	Retest (mean ± SD)	*p*	ICC – 3,1	SEM	SEM (%)	MD
1RM knee Extension (kg)	43.50 ± 14.20	45.90 ± 15.40	0.003	0.962	2.88	6.3	7.99
Isometric peak Torque (Nm)	159.02 ± 52.06	152.28 ± 49.75	0.152	0.878	17.76	11.4	49.22
Concentric peak torque (180°/sec; Nm)	77.35 ± 24.08	80.66 ± 28.21	0.161	0.884	8.92	11.3	24.73
Concentric peak torque (300°/sec; Nm)	67.41 ± 24.53	67.10 ± 26.36	0.945	0.541	17.25	25.7	47.81
Leg lean Mass – Bilateral (kg)	15.85 ± 3.76	15.93 ± 3.70	0.239	0.995	0.260	1.7	0.721
Leg lean Mass – Dominant (kg)	7.96 ± 1.94	8.00 ± 1.90	0.325	0.995	0.13	1.7	0.37
1RM knee extension/Bilateral leg lean Mass (kg/kg)	2.72 ± 0.56	2.87 ± 0.65	0.012	0.885	0.20	4.9	0.57
Isometric peak torque/Dominant leg lean Mass (Nm/kg)	19.85 ± 4.67	18.98 ± 4.72	0.116	0.779	2.08	10.7	5.77
Concentric peak torque/Dominant leg lean Mass (180°/sec/kg)	9.69 ± 1.91	10.10 ± 1.92	0.230	0.764	0.99	51.7	2.74
Concentric peak torque/Dominant leg lean Mass (300°/sec/kg)	8.40 ± 1.89	8.32 ± 1.97	0.857	0.163	1.76	21.1	4.90
VL CSA (cm^2^)	16.24 ± 5.13	16.50 ± 5.09	0.494	0.919	1.45	8.9	4.40
VL EI – Corrected (A.U.)	139.32 ± 39.24	140.85 ± 37.17	0.468	0.955	8.11	5.8	22.46
RF CSA (cm^2^)	8.31 ± 2.80	8.42 ± 2.76	0.564	0.931	0.73	9.5	2.02
RF EI – Corrected (A.U.)	131.22 ± 25.23	130.61 ± 23.28	0.933	0.950	5.30	5.1	14.72
TUG (sec)	6.69 ± 1.64	6.86 ± 2.16	0.001	0.800	0.44	6.5	1.23
Sit-to-stand (sec)	11.20 ± 3.08	10.99 ± 3.36	0.521	0.844	1.27	11.5	3.53

### SARC-F and KOOS

Only one participant in the high volume group was deemed to be at risk for sarcopenia based on the results from the SARC-F. The remaining participants were non-sarcopenic. The results from the KOOS survey are listed below in Table 2.

**TABLE 2:** Table depicting overall and subgroup KOOS scores for all completed participants.

KOOS score	Percentage (%), Mean ± SD
Overall	94.1 ± 7.2
Symptoms and stiffness subtotal	94.5 ± 7.4
Pain subtotal	95.9 ± 7.0
Function, daily living subtotal	97.9 ± 3.2
Quality of life subtotal	90.2 ± 13.9
Function, sports and recreational activities	91.9 ± 11.1

### Baseline differences between groups

At baseline, there were no statistically significant differences between the moderate and high volume resistance training groups for most outcome measures. Independent samples *t*-tests revealed no differences in VL EI (p = 0.835), RF EI (p = 0.528), VL CSA (p = 0.102), RF CSA (p = 0.972), bilateral leg lean mass (p = 0.510), or TUG performance (p = 0.218). Cohen’s *d* values for these variables were small to moderate (range = 0.01–0.76), with the largest effect seen for VL CSA (*d* = 0.76), favoring the high volume group. Notably, a significant group difference was observed for Chair Sit-to-Stand performance (p = 0.034, d = 0.94), with the high volume group demonstrating slower times at baseline. Although TUG did not differ significantly between groups, the effect size was moderate (d = 0.58), suggesting some baseline variability in functional performance.

### VL EI

**FIGURE 4:**
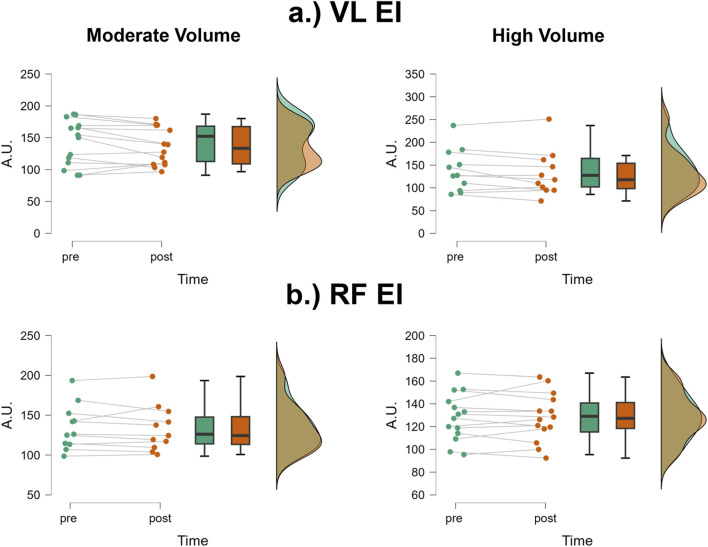
JASP raincloud plots showing group × time differences for **(a)** vastus lateralis (VL) and **(b)** rectus femoris (RF) echo intensity (EI).

The mean ± SD pretest and posttest VL EI values for the moderate volume group were 142.5 ± 35.7 A.U. and 136.2 ± 29.4 A.U., respectively, representing a 4.4% decrease (Figure 4a). For the high volume group, these corresponding values were 138.9 ± 46.7 A.U. and 131.7 ± 49.8 A.U., respectively, representing a 5.2% decrease. The number of participants that exceeded the MD were: moderate volume = 2/14, high volume = 1/11. The results from the two-way mixed factorial ANOVA indicated that there was no time × group interaction (F = 0.03, p = 0.874, η_p_^2^ = 0.001) and no main effect for group (F = 0.06, p = 0.803, η_p_^2^ = 0.003). There was, however, a significant main effect for time (F = 5.761, p = 0.025, η_p_^2^ = 0.200). When collapsed across group, the Bonferroni pairwise comparison indicated that VL EI significantly decreased by 4.8% following resistance training (pre and posttest marginal means = 140.7 and 133.9 A.U., respectively).

### RF EI

The mean ± SD pretest and posttest RF EI values for the moderate volume group were 128.3 ± 20.9 A.U. and 128.2 ± 21.2 A.U., respectively, representing only a 0.08% decrease (Figure 4b). For the high volume group, these corresponding values were 134.9 ± 28.5 A.U. and 133.5 ± 29.3 A.U., respectively, representing only a 1.04% decrease. The number of participants that exceeded the MD were: moderate volume = 1/14, high volume = 1/11. Of these two, both showed increases in RF EI following the training intervention. The results from the two-way mixed factorial ANOVA indicated that there was no time × group interaction (F = 0.168, p = 0.686, η_p_^2^ = 0.007), no main effect for group (F = 0.363, p = 0.553, η_p_^2^ = 0.02) and no main effect for time (F = 0.244, p = 0.626, η_p_^2^ = 0.01).

### VL CSA

**FIGURE 5:**
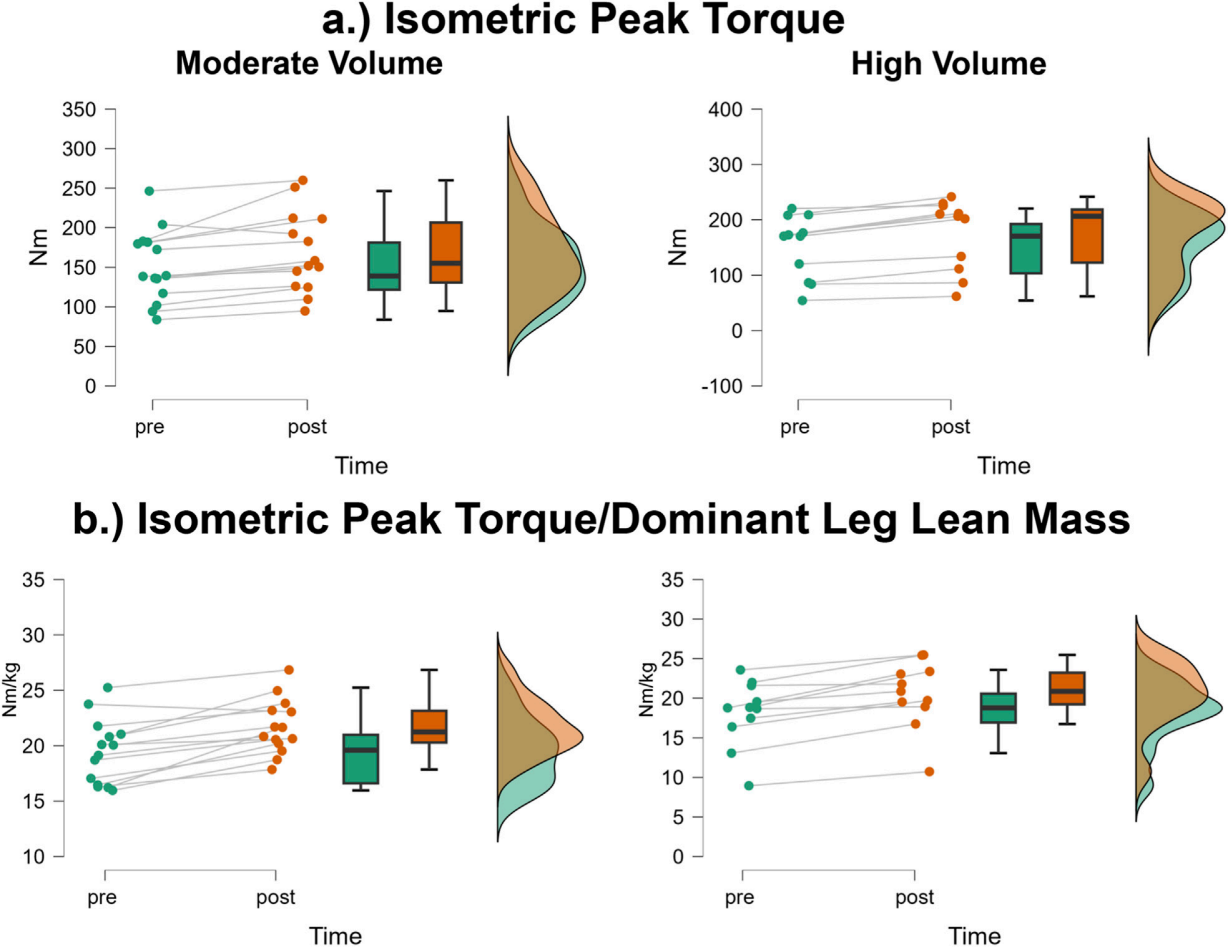
JASP raincloud plots showing group × time differences for **(a)** vastus lateralis (VL) and **(b)** rectus femoris (RF) cross-sectional area (CSA).

The mean ± SD pretest and posttest VL CSA values for the moderate volume group were 14.9 ± 3.58 cm^2^ and 16.6 ± 4.90 cm^2^, respectively, representing an 11.4% increase (Figure 5a). For the high volume group, these corresponding values were 18.9 ± 6.90 cm^2^ and 19.4 ± 6.7 cm^2^, respectively, representing a 6.0% change. The number of participants that exceeded the MD were: moderate volume = 2/14, high volume = 1/11. Of these 3, 1 participant from the moderate volume group showed a decrease, while the other two showed an increase. The results from the two-way mixed factorial ANOVA indicated that there was no time × group interaction (F = 0.367, p = 0.551, η_p_^2^ = 0.016) and no main effect for group (F = 2.012, p = 0.169, η_p_^2^ = 0.080). There was, however, a significant main effect for time (F = 6.95, p = 0.015, η_p_^2^ = 0.232). When collapsed across group, the Bonferroni pairwise comparison indicated that VL CSA significantly increased by 8.7% following resistance training (pre and posttest marginal means = 16.56 and 18.00 cm^2^).

### RF CSA

The mean ± SD pretest and posttest RF CSA values for the moderate volume group were 8.12 ± 2.28 cm^2^ and 8.89 ± 2.43 cm^2^, respectively, representing a 9.48% increase (Figure 5b). For the high volume group, these corresponding values were 8.08 ± 3.12 cm^2^ and 8.87 ± 3.48 cm^2^, respectively, representing a 9.78% increase. The number of participants that exceeded the MD were: moderate volume = 1/14 (increase), high volume = 0/11. The results from the two-way mixed factorial ANOVA indicated that there was no time × group interaction (F = 0.004, p = 0.950, η_p_^2^ < 0.001) and no main effect for group (F < 0.001, p = 0.981, η_p_^2^ < 0.001). There was, however, a significant main effect for time (F = 18.04, p < 0.001, η_p_^2^ = 0.440). When collapsed across group, the Bonferroni pairwise comparison indicated that RF CSA significantly increased by 9.6% following resistance training (pre and posttest marginal means = 8.10 and 8.88 cm^2^).

### Bilateral leg lean mass

The mean ± SD pretest and posttest bilateral leg lean mass values for the moderate volume group were 15.36 ± 3.73 and 15.43 ± 3.80 kg, respectively, representing only a 0.46% increase. For the high volume group, these corresponding values were 16.41 ± 4.01 kg and 16.56 ± 4.17 kg, respectively, representing a 0.91% increase. The number of participants that exceeded the MD were: moderate volume = 0/14, high volume = 2/11. Of these two, one showed an increase, whereas one showed a decrease. The results from the two-way mixed factorial ANOVA indicated that there was no time × group interaction (F = 0.224, p = 0.640, η_p_^2^ = 0.01), no main effect for group (F = 0.473, p = 0.499, η_p_^2^ = 0.02) and no main effect for time (F = 1.66, p = 0.210, η_p_^2^ = 0.07).

### Dominant leg lean mass

The mean ± SD pretest and posttest dominant limb leg lean mass values for the moderate volume group were 7.73 ± 1.94 and 7.76 ± 1.98 kg, respectively, representing only a 0.38% increase. For the high volume group, these corresponding values were 8.24 ± 2.03 kg and 8.30 ± 2.11 kg, respectively, representing a 0.73% increase. The number of participants that exceeded the MD were: moderate volume = 0/14, high volume = 2/11. Of these two, one showed an increase, whereas one showed a decrease. The results from the two-way mixed factorial ANOVA indicated that there was no time × group interaction (F = 0.103, p = 0.751, η_p_^2^ = 0.004), no main effect for group (F = 0.423, p = 0.522, η_p_^2^ = 0.02) and no main effect for time (F = 1.43, p = 0.244, η_p_^2^ = 0.06).

### 1RM knee extension

**FIGURE 6:**
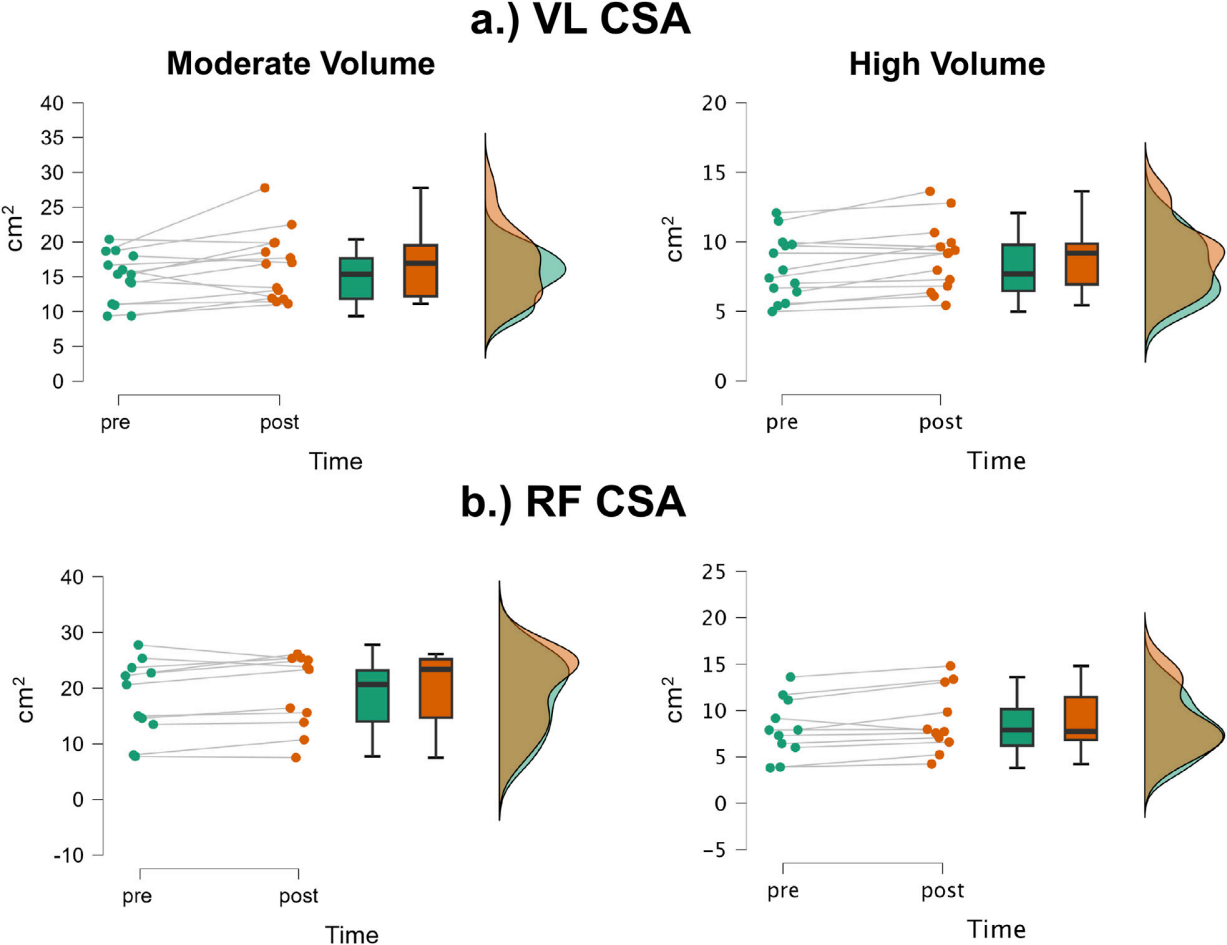
JASP raincloud plots showing group × time differences for **(a)** one-repetition maximum (1RM) knee extension and **(b)** 1RM knee extension/bilateral leg lean mass.

The mean ± SD pretest and posttest 1RM knee extension values for the moderate volume group were 43.9 ± 11.6 kg and 64.0 ± 18.7 kg, respectively, representing a 45.8% increase (Figure 6a). For the high volume group, these corresponding values were 47.0 ± 20.3 kg and 62.2 ± 25.6 kg, respectively, representing a 32.3% increase. The number of participants that exceeded the MD were: moderate volume = 13/14, high volume = 6/11. The results from the two-way mixed factorial ANOVA indicated that there was no time × group interaction (F = 1.14, p = 0.296, η_p_^2^ = 0.05) and no main effect for group (F = 0.008, p = 0.930, η_p_^2^ < 0.001). There was, however, a significant main effect for time (F = 60.25, p < 0.001, η_p_^2^ = 0.724). When collapsed across group, the Bonferroni pairwise comparison indicated that 1RM knee extension significantly increased by 38.8% following resistance training (pre and posttest marginal means = 45.44 and 63.08 kg, respectively).

### 1RM knee extension/bilateral leg lean mass

The mean ± SD pretest and posttest 1RM knee extension/bilateral leg lean mass values for the moderate volume group were 2.87 ± 0.39 kg/kg and 4.13 ± 0.57 kg/kg, respectively, representing a 43.9% increase (Figure 6b). For the high volume group, these corresponding values were 2.80 ± 0.840 kg/kg and 3.64 ± 1.03 kg/kg, respectively, representing a 30.0% increase. The number of participants that exceeded the MD were: moderate volume = 10/14, high volume = 10/11. The results from the two-way mixed factorial ANOVA indicated that there was no time × group interaction (F = 3.43, p = 0.077, η_p_^2^ = 0.130) and no main effect for group (F = 1.103, p = 0.305, η_p_^2^ = 0.046). There was, however, a significant main effect for time (F = 87.79, p < 0.001, η_p_^2^ = 0.792). When collapsed across group, the Bonferroni pairwise comparison indicated that 1RM knee extension relative to bilateral leg lean mass significantly increased following resistance training (pre and posttest marginal means = 2.83 and 3.88 kg).

### Isometric peak torque

**FIGURE 7:**
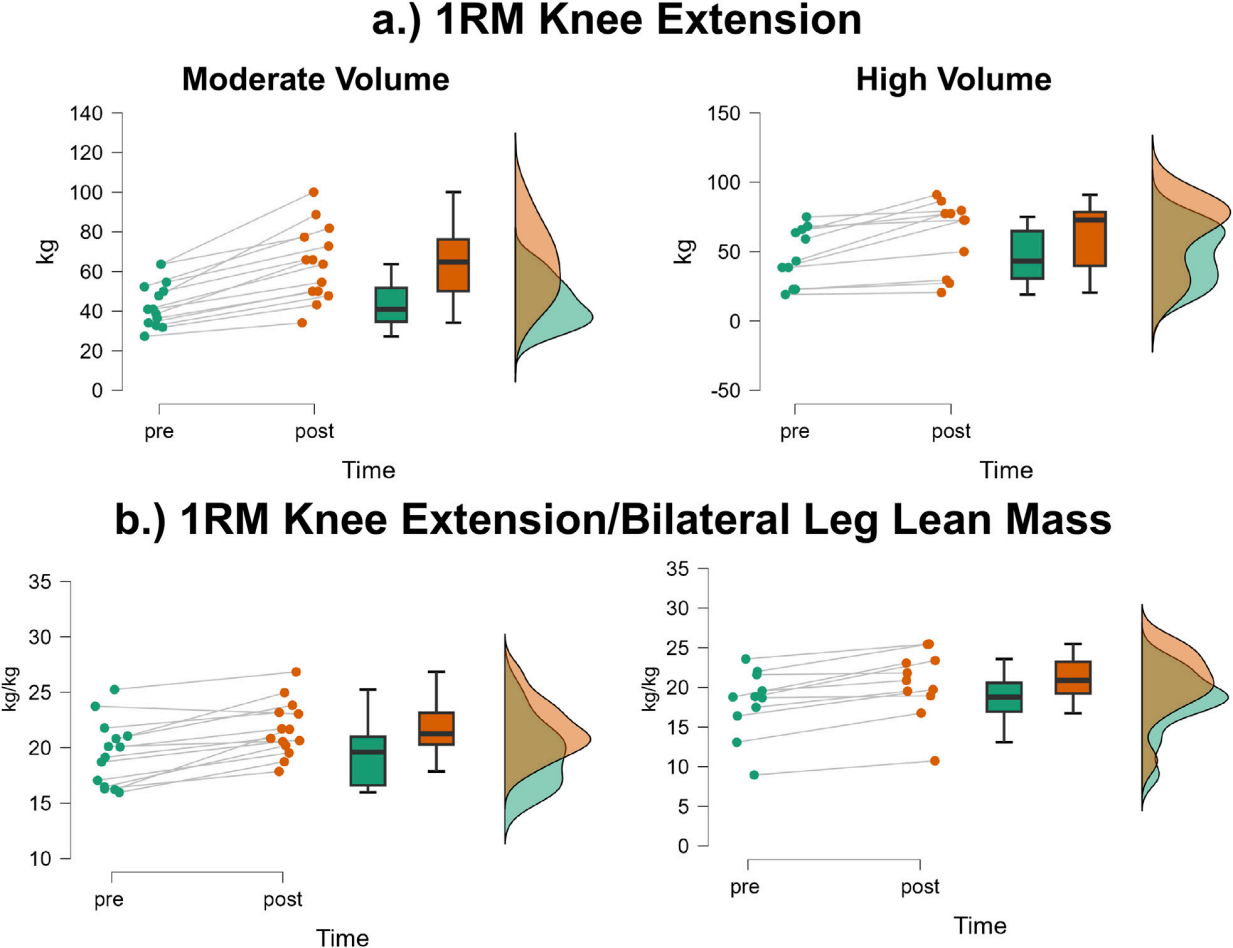
JASP raincloud plots showing group × time differences for **(a)** isometric peak torque and **(b)** isometric peak torque/dominant leg lean mass.

The mean ± SD pretest and posttest isometric peak torque values for the moderate volume group were 151.0 ± 45.7 Nm and 169.3 ± 50.79 Nm, respectively, representing a 12.1% increase (Figure 7a). For the high volume group, these corresponding values were 152.0 ± 56.7 Nm and 174.5 ± 63.7 Nm, respectively, representing a 14.8% increase. The number of participants that exceeded the MD were: moderate volume = 1/14 (increase), high volume = 0/11. The results from the two-way mixed factorial ANOVA indicated that there was no time × group interaction (F = 0.400, p = 0.533, η_p_^2^ = 0.017) and no main effect for group (F = 0.021, p = 0.886, η_p_^2^ < 0.001). There was, however, a significant main effect for time (F = 39.20, p < 0.001, η_p_^2^ = 0.630). When collapsed across group, the Bonferroni pairwise comparison indicated that isometric peak torque significantly increased by 13.5% following resistance training (pre and posttest marginal means = 151.5 and 171.9 Nm, respectively).

### Isometric peak torque/dominant leg lean mass

The mean ± SD pretest and posttest isometric peak torque values for the moderate volume group were 19.5 ± 2.9 Nm/kg and 21.7 ± 2.5 Nm/kg, respectively, representing an 11.3% increase (Figure 7b). For the high volume group, these corresponding values were 18.1 ± 4.16 Nm/kg and 20.5 ± 4.23 Nm/kg, respectively, representing a 13.3% increase. The number of participants that exceeded the MD were: moderate volume = 1/14, high volume = 0/11. The results from the two-way mixed factorial ANOVA indicated that there was no time × group interaction (F = 0.400, p = 0.533, η_p_^2^ = 0.017) and no main effect for group (F = 0.021, p = 0.886, η_p_^2^ < 0.001). There was, however, a significant main effect for time (F = 39.20, p < 0.001, η_p_^2^ = 0.630). When collapsed across group, the Bonferroni pairwise comparison indicated that isometric peak torque relative to dominant leg lean mass significantly increased by 12.2% following resistance training (pre and posttest marginal means = 18.8 and 21.1 Nm/kg, respectively).

### Concentric peak torque at 180°/second

The mean ± SD pretest and posttest concentric peak torque 180°/sec values for the moderate volume group were 76.7 ± 25.4 Nm and 83.3 ± 28.9 Nm, respectively, representing an 8.6% increase. For the high volume group, these corresponding values were 78.6 ± 26.6 Nm/kg and 83.6 ± 28.9 Nm/kg, respectively, representing a 6.4% increase. The number of participants that exceeded the MD were: moderate volume = 1/14, high volume = 0/11. The results from the two-way mixed factorial ANOVA indicated that there was no time × group interaction (F = 0.342, p = 0.564, η_p_^2^ = 0.015) and no main effect for group (F = 0.011, p = 0.917, η_p_^2^ = < 0.001). There was, however, a significant main effect for time (F = 16.84, p < 0.001, η_p_^2^ = 0.423). When collapsed across group, the Bonferroni pairwise comparison indicated that concentric peak torque at 180°/sec significantly increased by 7.6% following resistance training (pre and posttest marginal means = 77.6 and 83.5 Nm, respectively).

#### Concentric peak torque at 180°/second/dominant leg lean mass

The mean ± SD pretest and posttest concentric peak torque 180°/sec relative to dominant leg lean mass values for the moderate volume group were 17.3 ± 4.8 Nm/kg and 19.0 ± 4.5 Nm/kg, respectively, representing a 9.8% increase. For the high volume group, these corresponding values were 16.1 ± 4.6 Nm/kg and 17.6 ± 4.5 Nm/kg, respectively, representing a 9.3% increase. None of the participants showed changes that exceeded the MD (0/25). The results from the two-way mixed factorial ANOVA indicated that there was no time × group interaction (F = 0.094, p = 0.762, η_p_^2^ = 0.004) and no main effect for group (F = 0.511, p = 0.482, η_p_^2^ = 0.022). There was, however, a significant main effect for time (F = 10.85, p = 0.003, η_p_^2^ = 0.320). When collapsed across group, the Bonferroni pairwise comparison indicated that concentric peak torque at 180°/sec/dominant leg lean mass significantly increased by 9.6% following resistance training (pre and posttest marginal means = 16.7 and 18.3 Nm/kg, respectively).

### Concentric peak torque at 300°/second

The mean ± SD pretest and posttest concentric peak torque 300°/sec values for the moderate volume group were 61.6 ± 17.3 Nm and 66.0 ± 23.8 Nm, respectively, representing a 7.1% increase. For the high volume group, these corresponding values were 65.9 ± 19.6 Nm and 70.5 ± 21.4 Nm, respectively, representing a 7.0% increase. None of the participants showed changes that exceeded the MD (0/25). The results from the two-way mixed factorial ANOVA indicated that there was no time × group interaction (F = 0.003, p = 0.958, η_p_^2^ < 0.001) and no main effect for group (F = 0.296, p = 0.591, η_p_^2^ = 0.013). There was, however, a significant main effect for time (F = 8.19, p = 0.009, η_p_^2^ = 0.263). When collapsed across group, the Bonferroni pairwise comparison indicated that concentric peak torque at 300°/sec significantly increased by 7.1% following resistance training (pre and posttest marginal means = 63.8 and 68.3 Nm, respectively).

### Concentric peak torque at 300°/second/dominant leg lean mass

The mean ± SD pretest and posttest concentric peak torque 300°/sec relative to dominant leg lean mass values for the moderate volume group were 8.00 ± 1.13 Nm/kg and 8.42 ± 1.35 Nm/kg, respectively, representing a 5.3% increase. For the high volume group, these corresponding values were 8.00 ± 1.33 Nm/kg and 8.47 ± 1.35 Nm/kg, respectively, representing a 5.2% increase. None of the participants showed changes that exceeded the MD (0/25). The results from the two-way mixed factorial ANOVA indicated that there was no time × group interaction (F = 0.021, p = 0.885, η_p_^2^ < 0.001) and no main effect for group (F = 0.003, p = 0.961, η_p_^2^ < 0.001). There was, however, a significant main effect for time (F = 6.104, p = 0.021, η_p_^2^ = 0.210). When collapsed across time, the Bonferroni pairwise comparison indicated that concentric peak torque at 300°/sec relative to dominant leg lean mass significantly increased by 5.5% following resistance training (pre and posttest marginal means = 8.00 and 8.44 Nm/kg).

### Chair sit-to-stand

The mean ± SD pretest and posttest chair sit-to-stand values for the moderate volume group were 9.82 ± 3.01 s and 9.94 ± 3.28 s, respectively, representing a 1.2% increase. For the high volume group, these corresponding values were 12.92 ± 3.63 and 11.63 ± 3.12 s, respectively, representing a 10.0% improvement. The number of participants that exceeded the MD were: moderate volume = 1/14, high volume = 2/11. Of these two, one showed a decrease, whereas one showed an increase.

Given the presence of significant differences in Chair Sit-to-Stand times at baseline between the moderate and high volume groups, an ANCOVA was conducted to compare posttest scores while controlling pretest values. The pretest score was entered as a covariate, and group assignment served as the fixed factor. Results revealed a significant main effect of the covariate, indicating that baseline performance strongly predicted posttest values (F = 47.74, p < 0.001, ή^2^ = 0.685). After adjusting for baseline differences, there was no significant effect of training group on posttest Chair Sit-to-Stand performance (F = 0.96, p = 0.339, η_p_^2^ = 0.042).

### TUG

The mean ± SD pretest and posttest comfortable TUG values for the moderate volume group were 6.44 ± 1.00 s and 6.70 ± 1.29 s, respectively, representing a 4.0% increase. For the high volume group, these corresponding values were 7.77 ± 3.27 and 7.03 ± 1.58 s, respectively, representing a 9.52% improvement. The number of participants that exceeded the MD were: moderate volume = 0/14, high volume = 1/11 (faster time). The results from the two-way mixed factorial ANOVA indicated that there was no time × group interaction (F = 3.59, p = 0.07, η_p_^2^ = 0.135), no main effect for group (F = 1.133, p = 0.261, η_p_^2^ = 0.054), and no main effect for time (F = 0.813, p = 0.377, η_p_^2^ = 0.034).

### Dietary intake

All participants completed 3-day food logs, which were used to calculate their average daily intake. Independent samples t-tests revealed no significant differences between the moderate- and high-volume groups for any dietary variable:

- Calories: Moderate = 1900.5 ± 554.4 kcal, High = 1879.8 ± 606.3 kcal (p = 0.930, d = 0.036)
- Absolute protein: Moderate = 98.4 ± 25.5 g, High = 93.9 ± 26.2 g (p = 0.672, d = 0.173)
- Relative protein/bodyweight (kg): Moderate = 1.40 ± 0.45 g/kg, High = 1.23 ± 0.23 g/kg (p = 0.306, d = 0.422)
- Carbohydrates: Moderate = 186.3 ± 66.2 g, High = 217.6 ± 56.6 g (p = 0.225, d = 0.503)
- Fat: Moderate = 82.4 ± 32.0 g, High = 77.4 ± 35.1 g (p = 0.712, d = 0.150)

### Physical activity

All participants wore an accelerometer for seven consecutive days to assess physical activity. Independent samples t-tests showed no significant differences between groups:

- Moderate-to-vigorous physical activity:
o Moderate group: 37.1 ± 42.2 min/day
o High group: 28.0 ± 16.8 min/day
o p = 0.510, d = 0.270
- Step count:
o Moderate group: 5689.7 ± 3906.5 steps/day
o High group: 5774.2 ± 3906.5 steps/day
o p = 0.955, d = 0.023

Four total days were excluded from the analysis due to participants not meeting the minimum 10-h wear time threshold.

## Discussion

Studies among older adults attempting to identify improvements in EI following resistance training have shown mixed results, which may be due to differences in resistance training protocols, particularly as it relates to volume. Resistance training guidelines for older adults recommend 1–3 sets per exercise of compound, multi-joint exercises (Fragala et al., 2019). However, due to interindividual response heterogeneity and non-responders to exercise, higher training volumes may be needed to elicit favorable changes among older adults (Abrahin et al., 2014; Lixandrão et al., 2024). Therefore, the present exploratory study aimed to compare the effects of 6 weeks of moderate and high volume resistance training on various measures of muscle size, strength, and quality among older adults. Our main finding was that higher training volume did not lead to significantly greater improvements across any of the measured outcomes compared to moderate training, suggesting that higher volume may not be necessary to achieve beneficial results. Furthermore, the results of the present study show a significant decrease in corrected EI for the VL, with no meaningful difference in RF EI, but increases in both VL and RF CSA. As expected, resistance training led to significant improvements in knee extension 1RM, as well as isometric and concentric peak torque in both groups. Functional test performance showed non-significant changes, with slightly slower times in the moderate volume group but small improvements in the high volume group. Below, we discuss the implications, strengths, and limitations of these findings.

### EI

Our data shows a significant decrease in corrected VL EI regardless of training volume, which could be attributed to improved intramuscular adiposity within the skeletal muscle (Reimers et al., 1993; Young et al., 2015). Interestingly, we observed no significant improvements in corrected RF EI, suggesting a muscle specific response to the training protocol. Our findings contrast with Radaelli et al. (2019), who observed improvements in quadriceps EI (i.e., RF + vastus intermedius + vastus medialis + VL/4), performing 1 or 3 sets for 12 weeks. However, improvements were not similar within groups, as greater improvements in vastus medialis EI were observed with 1 set per exercise, while the RF EI improved with 3 sets. Similarly, Radaelli et al. (2013), Radaelli et al. (2014a) observed that following 13 or 20 weeks of progressive resistance training, RF EI significantly improved regardless of performing 1 or 3 sets per exercise. The decrease in VL EI, but not RF EI, observed herein could be due to the specific nature of our training protocol. The use of our specific exercises in training might have led to greater activation of the VL (Da Silva et al., 2008; Camara et al., 2016). While significant improvements in EI were observed in the aforementioned studies, the differences in training protocols are noteworthy. We utilized a high intensity (>85% 1RM, <8 repetitions) training intervention for 6 weeks, while prior investigations employed a lower intensity (<85% 1RM) training intervention for ≥12 weeks (2013, 2019). It is plausible that the high intensity nature of our training protocol was effective for eliciting maximal strength improvements in a short period of time, and a longer training period may be required to elicit uniform changes in quadriceps EI. Interestingly, our group observed contrasting findings to changes in EI following resistance training, performing the same protocol as the present study, with the exception of the lying leg curl (Knowles et al., 2025). In that study, we observed significant improvements in RF EI, but not VL EI, following 6 weeks of high intensity lower body resistance training. However, it is important to note that work was a secondary analysis, and the sample population consisted of only 12 older adults, mainly female. Therefore, it may be difficult to generalize the findings from Knowles et al. (2025) to other investigations with a more complete sample. Future researchers examining changes in EI following resistance training should consider the mechanisms behind non-uniform changes in quadriceps EI, in the presence of different training volumes.

### Maximal quadriceps strength

Unsurprisingly, resistance training led to significant improvements in maximal quadriceps strength across both training volume groups. This general pattern was observed across multiple strength assessment modes; however, gains in 1RM knee extension strength were notably larger than those seen in unilateral isometric and concentric peak torque. This discrepancy is likely due to task specificity, as the training more closely resembled the movement and loading pattern of the 1RM test. Our findings are consistent with a number of studies showing that, during the initial phases of resistance training (i.e., the first 6–12 weeks), the number of sets performed has minimal impact on maximal strength gains, provided that a basic threshold of training volume is met (Radaelli et al., 2013; Scanlon et al., 2014; Pagan et al., 2024). For example, Radaelli et al. (2013) reported a 31.8% and 38.3% increase in knee extension 1RM performing either 1 or 3 sets for 13 weeks, respectively. Additionally, Galvão and Taaffe (2005) observed similar results, as they found a 20.8% and 38.9% increase in knee extension 1RM performing either 1 or 3 sets, respectively, for 20 weeks. These findings suggest a point of diminishing returns for strength gains among older adults. In fact, it is interesting to note that, despite no significant time × group interaction, 1RM strength improvements were slightly better for the moderate volume training group. Specifically, 1RM knee extension strength increased by 45.8% in the moderate volume group and 32.3% in the high volume group, and nearly all the participants in the moderate group (13 of 14) exceeded the MD, compared to only 6 of 11 in the high volume group. An important consideration not fully addressed in this study is the possibility that individuals in the high volume group experienced impaired recovery, which may have limited their adaptive response to training. As noted by Li et al. (2024), older adults may be more vulnerable to muscle damage and require longer recovery periods due to age-related declines in satellite cell availability, elevated oxidative stress, reduced muscle excitation, and diminished muscle protein synthesis. Taken together, our results further support the use of minimal effective training volumes for older adults participating in resistance training, especially given the reduced recovery capacity commonly associated with aging (Borde et al., 2015).

### Muscle size

Similar to the improvements observed in maximal strength, we found that both VL and RF CSA increased regardless of training volume. This suggests that higher training volumes may not be necessary to elicit hypertrophic adaptations during the early phases of resistance training in older adults. These results align with several previous studies that have reported increases in muscle size among older adults following resistance training intervention (Radaelli et al., 2013; Scanlon et al., 2014; Pagan et al., 2024). However, although significant increases in muscle CSA were detected using ultrasonography, no corresponding changes were observed with BIA, as leg lean mass remained unchanged. These results are in agreement with many studies that have reported dissociations between measures of individual muscle size and whole limb or body segment lean mass despite different methodological approaches (Scanlon et al., 2014; Tavoian et al., 2019; Ruple et al., 2022). For example, following 6 weeks of resistance training among older adults, Scanlon et al. (2014) reported significant increases in VL CSA, but thigh lean mass assessed with dual energy X-ray absorptiometry (DXA) did not improve. (2022) observed a similar phenomenon as they measured hypertrophic responses using common assessments following 10 weeks of resistance training among healthy adults and found magnetic resonance imaging and DXA to detect significant increases in muscle size, but ultrasound derived VL CSA did not. However, ultrasound was in good agreement with magnetic resonance imaging for tracking changes. These results, along with our own findings, suggest that ultrasound may be more sensitive to detecting localized changes in muscle size, whereas body composition methods such as BIA offer broader, less sensitive assessments of leg lean mass that may not reflect short-term training adaptations. Researchers should consider these differences when designing clinical trials to evaluate muscle size changes in older adults, as the choice of measurement technique can influence the interpretation of outcomes.

### Quadriceps functional muscle quality

Given that the term muscle quality often refers to both tissue composition and force-generating capacity relative to muscle size, a sub-aim of this study was to examine changes in maximal strength relative to leg lean mass. As we found that neither moderate nor high volume resistance training increased leg lean mass, normalization of our measures of maximal strength (i.e., 1RM knee extension, isometric and concentric peak torque) did not offer novel findings beyond reporting them alone. Despite differing approaches, our study results are in agreement with others that have examined changes in maximal strength relative to muscle size in conjunction with assessments of EI in both younger (Mota et al., 2017) and older populations (Radaelli et al., 2013; Scanlon et al., 2014). This contrast highlights that different measures of muscle quality may respond differently to short-term resistance training and should be interpreted independently. Tissue composition and strength-to-size ratios reflect distinct physiological constructs, so relying on a single measure may provide an incomplete understanding of how muscle quality adapts in older adults. It is important to note that the short duration of this study, combined with the focus on moderate-load training aimed at improving maximal force output, may have favored strength gains that outpaced muscle hypertrophy. Further research with longer interventions is needed to identify resistance training protocols that effectively promote concurrent improvements in muscle strength, size, and tissue composition (as reflected by EI).

### Functional outcomes

Perhaps the most unique finding of the present study was the divergent, though non-significant, changes in functional test performance between the moderate and high volume groups. Moderate effect sizes were observed for the group × time interactions for both functional measures, suggesting meaningful, albeit non-significant, trends. The high volume group demonstrated the greatest improvements, showing gains of ≥9.5% in both functional tests. In contrast, the moderate volume group experienced slight declines in performance, as reflected by increased test completion times. While it may be reasonable to suggest that higher training volumes are necessary to improve functional performance in older adults, it is important to note that the training protocols in this study did not specifically target the movement patterns or task demands of the TUG or Sit-to-Stand tests. Recent work by Pagan et al. (2024) underscores the importance of task-specific training for improving functional performance in older adults. In their study, participants who engaged in traditional resistance training demonstrated significant improvements in 5RM for the same exercises used during training, but showed little to no gains in functional performance tests. In contrast, those who performed functional resistance training exhibited the opposite pattern—limited improvements in traditional strength measures but significant gains in functional tasks. These findings suggest that while high volume resistance training may lead to some transfer to non-specific tasks, more targeted, task-specific training may be necessary to optimize improvements in tests such as the TUG and Sit-to-Stand. One aspect of our own functional test data merits clarification. Of all participants in our completed dataset, only one was classified as sarcopenic based on the SARC-F questionnaire; this individual was randomized to the high volume training group. At baseline, they experienced extreme difficulty completing the functional tests, but by the end of the intervention, they performed both tasks with little to no difficulty. Notably, this participant was also one of the few to exceed the MD for both tests, highlighting the potential for clinically relevant functional improvements among older adults with lower baseline capacity.

### Strengths and limitations

Our exploratory study had multiple strengths that should be considered when contextualizing our findings. First, our study was strengthened by a experimental design featuring several testing controls (i.e., testing and training at the same time of day, qualified supervision for all visits, hydration assurance, the ultrasound investigator was blinded, concern for nutrition, etc.). All 25 participants attended all training and testing sessions and were thoroughly familiarized with testing and training protocols. We also established unique, sample specific reliability statistics from the first two pretesting sessions, which allow us to report how meaningful each participant’s change was. We also used a training frequency of twice/week, as this was shown to provide favorable adaptations among older adults (Fragala et al., 2019). Nevertheless, our study had several limitations that should be acknowledged. First, the sample consisted of generally healthy older adults without major physical impairments, which limits the generalizability of our findings to frail or sarcopenic populations. Second, the training protocol focused exclusively on lower body resistance exercises and did not incorporate upper body or aerobic training. We also did not monitor or restrict participants from engaging in other forms of physical activity outside the laboratory, which could have influenced the outcomes. Additionally, although our inclusion criteria excluded individuals who had performed lower body resistance training within the past 6 months, several participants had previous resistance training experience and used the study as an opportunity to reestablish a consistent training routine. This retraining effect in a subset of the sample may have contributed to more rapid improvements in strength and muscle size, potentially inflating the observed effects. Thirdly, we did not include the use of a time matched non-exercise control group in our study, which would’ve bolstered the study design. The use of a time matched non-exercise control group is often recommended in exercise science research, particularly in training interventions for naïve trainees, as it may be difficult to attribute improvements solely to the intervention (Hammert et al., 2024). Moreover, our study duration of only 6 weeks was short compared to other previous long term studies. Due to the short nature of the study, our findings, primarily as it relates to strength could have been due to neural adaptations. Finally, our study did not include advanced assessments of neural function, such as transcranial magnetic stimulation, motor unit analysis, or peripheral nerve stimulation. While this limits our ability to draw conclusions about neuromuscular mechanisms underlying the observed adaptations, it also highlights opportunities for future research. Considering both the strengths and limitations of our approach can help inform the design of more comprehensive studies moving forward.

## Summary

In summary, the key finding from this study is that higher volume resistance training did not result in greater improvements in muscle strength, size, or quality compared to moderate volume training, based on a comprehensive set of outcome measures. Both training volumes were effective in improving maximal strength and increasing VL and RF CSA. However, the extent of these improvements varied by outcome and muscle group. For example, VL EI, a marker of muscle tissue composition, improved regardless of training volume, while RF EI did not show significant change. Additionally, although CSA increased, leg lean mass assessed by BIA remained unchanged, highlighting discrepancies between imaging-based and body composition methods. These findings suggest that short-term training adaptations may be more easily detected through localized measures such as ultrasound rather than whole-limb assessments. The lack of added benefit from higher volume also supports the efficiency and practicality of simple, time-efficient, and moderate volume programs for older adults. Future research should investigate the effects of long-term training and the inclusion of task-specific or multimodal exercise to enhance functional outcomes.

## Data Availability

The raw data supporting the conclusions of this article will be made available by the authors, without undue reservation.
